# Assessing barriers to access and equity for COVID-19 vaccination in the US

**DOI:** 10.1186/s12889-022-14636-1

**Published:** 2022-12-03

**Authors:** Michael Kuehn, Joyce LaMori, Jessica K. DeMartino, Marco Mesa-Frias, Jason Doran, Lohit Korrapati, Rhea Bhojwani, Patrick Lefebvre, Noam Kirson

**Affiliations:** 1 Analysis Group, Inc., 151 W 42nd Street, 23rd floor, New York, NY USA; 2 Janssen Scientific Affairs, LLC, Titusville, NJ USA; 3 Analysis Group, Inc., Menlo Park, CA USA; 4 Analysis Group, Inc., San Francisco, CA USA; 5 Groupe d’analyseLtée, Montréal, QC Canada; 6 Analysis Group, Inc., Boston, MA USA

**Keywords:** Preventive Medicine, Population Health, Health Equity, Health Disparities, Community Health

## Abstract

**Background:**

Historical vaccination coverage in economically disadvantaged, ethnic minority, non-affluent white and agricultural populations in the US has lagged coverage in more affluent urban and suburban white populations due to a variety of social and economic factors. In the current COVID-19 pandemic, sociocultural and economic challenges continue to present significant obstacles to achieving equitable uptake of COVID-19 vaccines. The goal of this study was to qualitatively assess perceptions of key US healthcare stakeholders of the most significant barriers to COVID-19 vaccine access and equity to better characterize their expected impact on US communities.

**Methods:**

After conducting a targeted literature review (TLR), we hypothesized 20 high-impact barriers which included structural and logistical barriers, capturing systemic challenges to vaccine accessibility, and attitudinal and informational barriers, affecting patient willingness to pursue vaccination. We developed a qualitative discussion guide, which included both open-ended and closed-ended questions, and interview stimulus material to conduct one-on-one in-depth interviews to assess the expected prevalence, severity, and persistence of these 20 high-impact barriers, which were hypothesized based on TLR. As a part of this qualitative study, we conducted one-on-one in-depth interviews with a diverse set of 15 US healthcare stakeholders who were involved in the COVID-19 vaccine rollout in states with relatively disparate vaccination rates by ethnicity. These stakeholders were selected to reflect an array of roles in the COVID-19 vaccine rollout, including infectious disease specialists, pharmacists, community advocacy representatives, and partners of local governments involved in the COVID-19 vaccine rollout and community education.

**Results:**

Respondents identified limited vaccination sites in rural settings and technology-related barriers as the most prevalent and severe structural and logistical barriers in US communities. Respondents assessed COVID-19 vaccine safety concerns and politically motivated skepticism to be the most prevalent and severe attitudinal and informational barriers. Respondents cited proliferation of mobile vaccination clinics and local community messaging to endorse vaccines as the most effective solutions to these top structural and attitudinal barriers. Respondents expected politically motivated skepticism to be the most significant and persistent barrier to broader vaccine uptake in the US.

**Conclusions:**

Our study suggests that attitudinal barriers, particularly politically motivated skepticism, are likely to remain the most persistent challenges to widespread vaccination against COVID-19 in the US.

## Background

As of August 23, 2022 the Centers for Disease Control and Prevention (CDC) reported that 90% of the US population aged 18 years and older had received at least one dose of a COVID-19 vaccine [1]. While the rate of COVID-19 vaccination in the US accelerated in the first few months of 2021, the pace of new patients receiving doses has slowed since April.

Existing literature on historical vaccination programs in the US reflects consistently low vaccination rates among socially and economically disadvantaged, ethnic minority, and rural communities due to a variety of structural and attitudinal challenges [2–5]. Indeed, despite universal COVID-19 vaccine availability in the US, disparities in vaccination rates persist, and remain particularly pronounced in certain US subpopulations and locations [6]. CDC reports that the COVID-19 vaccination rate for African Americans has lagged that of the general US population [7]. Furthermore, survey data from the Kaiser Family Foundation (KFF) show that rural residents and white evangelical Christians continue to lag the general US adult population in uptake of COVID-19 vaccines [6].

Improving characterization of barriers to equity and access for COVID-19 vaccination will enable decision-makers to adjust patient outreach and care delivery strategies to minimize disparities in COVID-19 vaccination rates among lagging and difficult-to-reach patient communities. The goal of this study was to qualitatively assess key stakeholders’ perceptions of the most significant barriers to COVID-19 vaccine access and equity to better characterize the variation in their impact on US communities and to hypothesize potential solutions to the most persistent and disruptive challenges.

## Methods

First, we conducted a targeted literature review (TLR) in late March/early April 2021 to inform hypotheses on the most prevalent and impactful equity and access barriers. To capture both historically persistent barriers to vaccination in the US and new barriers stemming from the COVID-19 pandemic, we conducted a two-step TLR which included peer-reviewed literature and non-peer-reviewed literature. The peer-reviewed portion of the TLR was conducted using the OvidSP database, with the goal of identifying key barriers to access and equity observed in the US in historical adult vaccination programs prior to COVID-19. The non-peer-reviewed portion of the TLR was conducted to capture the ongoing developments in the COVID-19 vaccination landscape given the lag time anticipated for current research publications. In total, 70 articles (29 peer-reviewed, 41 non-peer-reviewed articles) were selected and reviewed to inform initial hypotheses on the most significant barriers to equity and access for COVID-19 vaccines.

Following the TLR, a qualitative discussion guide, which included both open-ended and closed-ended questions, and interview stimulus material were developed and refined through two pilot interviews with infectious disease experts to prepare for research interviews. Discussion materials were designed to solicit participants’ perceptions of current and future barriers to access and equity for COVID-19 vaccination in the US. We then identified and recruited COVID-19 vaccine stakeholders for one-on-one in-depth interviews to assess the observed impact of, and potential solutions for, the hypothesized barriers. First, we identified the states with pronounced gaps in COVID-19 vaccination rates between Caucasians and African American/Hispanic populations. Based on the disparities in vaccination by ethnicity at the state level, we then directed recruitment to stakeholders in Arizona, Colorado, Connecticut, Florida, Georgia, New York, North Carolina, Pennsylvania, and Wisconsin [8]. To capture a variety of perspectives on disparities and barriers that exist in local communities from an array of professionals involved in local COVID-19 vaccine rollout and community education, our study sample of 15 experts included 4 infectious disease specialists, 2 independent pharmacists, 2 large-chain pharmacists, 2 community advocacy representatives, and 5 partners of local governments from the prioritized states. These stakeholders spanned a range of functions in local COVID-19 vaccine distribution and uptake, including vaccination, program administration, rollout and education, and were selected to provide a broader perspective on their local vaccination programs beyond the point of care.

In the expert interviews conducted between April 14, 2021 and May 4, 2021, the respondents were first asked open-ended questions about their perception of the COVID-19 vaccine rollout and barriers to access and equity for COVID-19 vaccines that they had observed, either in their practices or localities. They were then shown the list of hypothesized barriers from the TLR and were asked to assess both the prevalence of the barrier across US communities and the severity of the barrier’s impact within affected communities for each of the hypothesized barriers on a 5-point scale and provide rationale for their ratings. Barriers were then ranked during the interviews using a composite score based on the average of their rated prevalence and severity scores to facilitate deep dives into barriers with the highest impact on access to COVID-19 vaccines in their localities. For each of the top-ranked three barriers, the respondents were asked to elaborate on which communities had been disproportionately impacted, potential solutions, and their expected persistence over time. The deep dives were guided by the initial unprompted responses of the respective expert around access and equity barriers as well as the structured responses to perceived prevalence and severity. In the data analysis, we coded expert responses to open-ended questions on topics such as potential solutions and COVID-19 vaccine attributes to mitigate barriers to access and equity and aggregated qualitative responses to provide context to the responses to structured questions.

## Results

### Hypothesized high-impact barriers grouped into structural and attitudinal challenges to vaccine access and equity

Based on the targeted literature review, 20 high-impact barriers to COVID-19 vaccine access and equity were prioritized for testing in discussions with healthcare delivery, outreach, and advocacy stakeholders. While these 20 barriers are not considered mutually exclusive and are subject to overlapping influence, we categorized them into 2 groups for stakeholder testing: (1) structural and logistical barriers, defined as barriers primarily driven by systemic challenges to vaccine supply or accessibility, and (2) attitudinal and informational barriers, defined as barriers primarily driven by information ecosystem and decision-making patterns that affect patient willingness to pursue vaccination. Structural and logistical barriers discussed with COVID-19 vaccine stakeholders included:

1. Underinsurance or lack of health insurance
2. Lack of preventative care access
3. Delayed care-seeking behavior
4. Provider expectation that vaccine acquisition or administration will not be sufficiently reimbursed
5. Inadequate staffing or supplies at vaccination sites
6. Inadequate immunization information or vaccine management systems
7. Limited vaccination sites in rural settings
8. Inadequate access to transportation
9. Technology-related barriers (e.g., issues with scheduling appointments online)
10. Work or childcare requirements that limit schedule flexibility

Attitudinal and informational barriers discussed with COVID-19 vaccine stakeholders included:

1. Hesitancy around unfamiliar vaccination setting (e.g., mass vaccination sites)
2. Medical mistrust
3. Lack of eligibility awareness, including complexity of eligibility tiering system
4. Primary news consumption via social media and politically motivated skepticism
5. Concerns about the COVID-19 vaccine (e.g., safety)
6. Vaccine hesitancy or lack of familiarity with vaccines
7. Low familiarity with healthcare system and/or delivery points
8. Language barriers (limited ability to speak/understand English)
9. Fear of registration for follow-ups for multi-dose vaccines
10. Perceived underrepresentation of minorities in COVID-19 vaccine clinical trials

### COVID-19 vaccine safety concerns and populations in remote settings represent substantial barriers

Prior to reviewing the list of 20 hypothesized barriers, participants were first asked to name the main barriers to access and equity for COVID-19 vaccines that they have observed in their communities. The three most frequently cited barriers were: COVID-19 vaccines safety concerns, general vaccine hesitancy and technology-related barriers. As the interviews were conducted in late April and early May, respondents noted that the temporary pause of the Janssen COVID-19 vaccine announced by the FDA on April 13, 2021 further increased COVID-19 vaccine safety concerns [9].

**Fig. 1:**
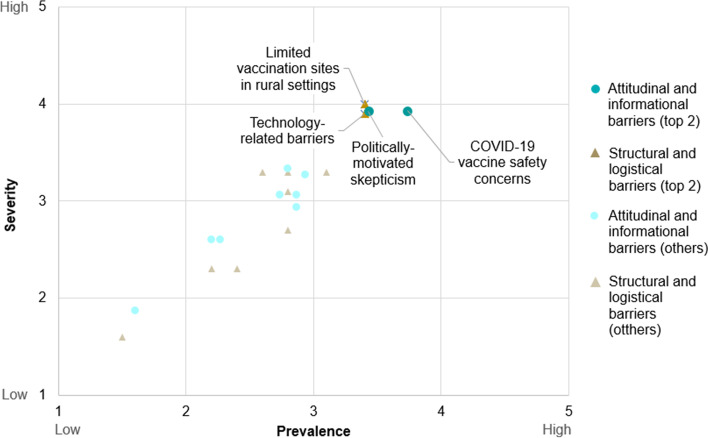
Top rated barriers to COVID-19 vaccine access and equity by prevalence and severity (average ratings). Top rated barriers to COVID-19 vaccine access and equity by prevalence and severity (average ratings, *n* = 15 interviews)

Participants were then asked to rate the prevalence and severity of each of the 20 barriers and to provide a rationale for their ratings. Based on composite scoring of prevalence and severity the top 2 rated *structural and logistical* barriers were: limited vaccination sites in rural settings and technology-related barriers (see Fig. 1). The top 2 rated *attitudinal and informational* barriers were: COVID-19 vaccine safety concerns and politically motivated skepticism (see Fig. 1). Among all 20 hypothesized barriers tested, stakeholders rated concerns about the safety of COVID-19 vaccines to have the highest detrimental impact on COVID-19 vaccination access and equity.

### Top structural and logistical barriers noted underscore the impact of social vulnerability on limiting access to COVID-19 vaccines

**Fig. 2:**
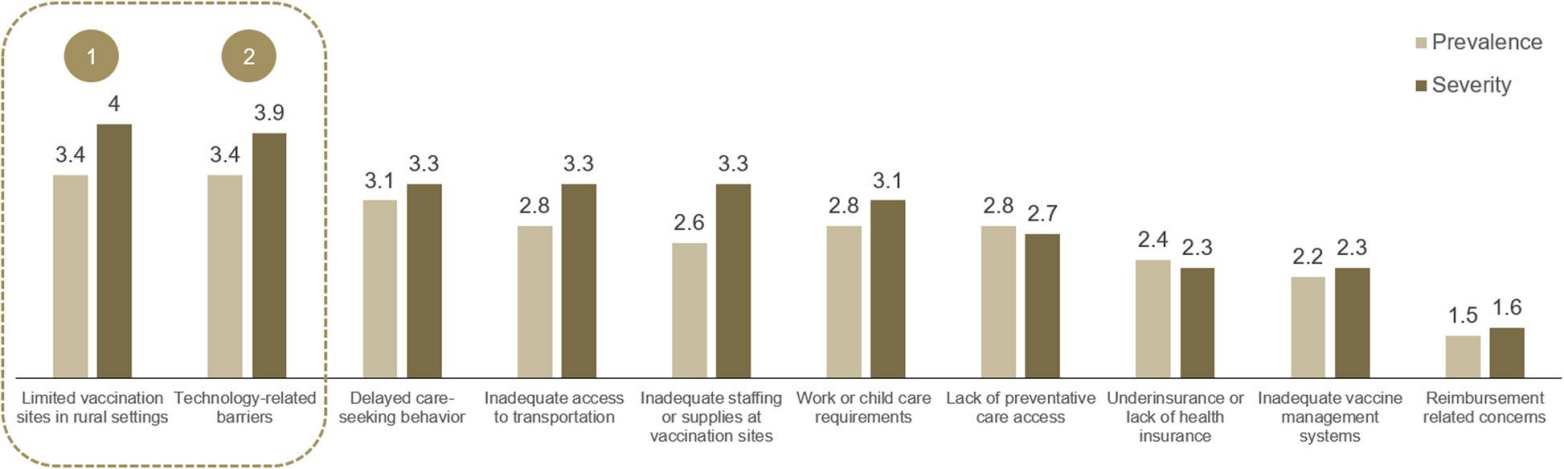
Prevalence and severity of structural and logistical barriers (average ratings, *n* = 15 interviews)

Within structural and logistical barriers, respondents considered the impact, as determined by the combined prevalence and severity ratings, of limited vaccination sites in rural settings and technology-related barriers to be higher than that of other structural and logistical barriers (see Fig. 2).

Respondents considered migrant farm workers, elderly, and middle-class, agricultural Caucasian communities to be disproportionately affected by limited vaccination sites in rural settings relative to other rural communities. Low vaccination rates in these communities were considered likely to be further compounded by inadequate access to transportation and politically motivated skepticism. The most frequently cited potential solutions to improve vaccination rates in these vulnerable rural communities were related to the creation of mobile vaccine initiatives to increase vaccination convenience. These initiatives included vaccination events at patients’ places of work (farms, factories etc.) and administration of vaccines at patients’ homes. The latter was considered particularly important for reaching patients who are elderly and homebound or who lack access to transportation (see Table 1).

**Table 1:** Top 2 structural and logistical barriers identified from 15 interviews

	**Limited vaccination sites in rural settings**	**Technology-related barriers**
Subpopulation(s) most affected	▪ Migrant farm workers with insufficient access to transportation ▪ Middle-class, agricultural Caucasian communities ▪ Elderly in rural areas	▪ Elderly patients ▪ Hispanic and African American communities ▪ Immigrant/refugee communities ▪ Low/middle income populations
Related or overlapping barriers	▪ Inadequate access to transportation ▪ Politically motivated skepticism	▪ Inadequate access to transportation
Potential solutions	▪ Vaccination events at places of work (e.g., farms, factories) ▪ Administering vaccines directly at the homes of harder-to-reach populations such as elderly in rural areas	▪ Increase the number of walk-in and mobile clinics ▪ Alternative appointment scheduling, such as contacting patients proactively by phone or home-visit appointments ▪ Increase availability of COVID-19 vaccines in traditional healthcare delivery settings (e.g., primary care physician offices)

Respondents expected technology-related barriers (e.g., issues with scheduling appointments online) to disproportionately affect elderly, Hispanic, African American, immigrant/refugee and low and middle-income communities. Despite the wide-spread impact, technology-related barriers were expected by respondents to resolve relatively quickly as vaccine supply continues to grow and appointment-scheduling becomes less dependent on online platforms. Respondents noted that increasing the number of mobile and walk-in clinics, and more proactive outreach to patients, including contacting patients by phone and home-visit appointments, as well as increasing availability of COVID-19 vaccines in traditional healthcare delivery settings (e.g., primary care physician offices) may help address the challenges posed by technology-related barriers (see Table 1).

Respondents rated reimbursement-related concerns as the least significant barrier tested as most believed providers felt assured of adequate universal coverage of product and administration. However, independent pharmacists were a notable exception, noting that delayed or inadequate coverage could present a significant challenge given the occasionally disruptive investment and opportunity costs (personnel training, storage, administration etc.) required to provide COVID-19 vaccines. As independent pharmacies are often key sites of care for low-income and rural populations, these concerns could exacerbate vaccination disparities in these vulnerable communities if coverage for future booster vaccines is insufficient [10].

### Politically motivated skepticism is considered the single most challenging barrier to overcome in the long run

**Fig. 3:**
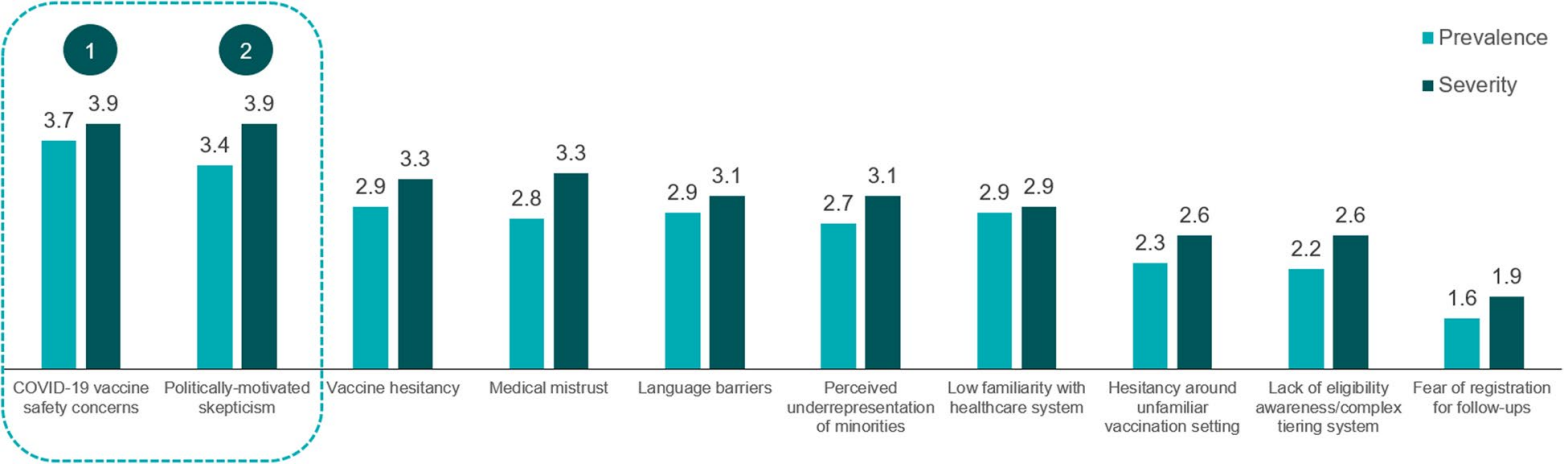
Prevalence and severity of attitudinal and informational barriers (average ratings, *n* = 15 interviews)

Among attitudinal and informational barriers, experts identified politically motivated skepticism to have the highest impact on unequal COVID-19 vaccine access and equity (see Fig. 3).

COVID-19 vaccine safety concerns were considered to be the most prevalent barrier to COVID-19 vaccination access and equity, affecting a wide array of communities including African American, Hispanic, immigrant and refugee communities, people with low educational attainment, elderly patients, and politically and religiously conservative populations. Respondents mentioned that affected communities tended to express concerns about the speed of vaccine development, novel mechanisms of action, observation of burdensome side effects, and the spread of misinformation on unsubstantiated health consequences of COVID-19 vaccination. Despite being considered a relatively severe near-term barrier, stakeholders were optimistic that COVID-19 vaccine safety concerns will resolve naturally in a large subset of concerned populations as more patients continue to receive vaccinations with limited adverse events and skeptical patients who prefer a “wait and see” approach gain comfort with the vaccines. Respondents also noted that partnerships with local leaders and community-based organizations, with emphasis on endorsement and promotion of COVID-19 vaccines by local healthcare providers, will be essential to increasing vaccination rates among these communities (see Table 2).

**Table 2:** Top 2 attitudinal and informational barriers identified from 15 interviews

	**COVID-19 vaccine safety concerns**	**Primary news consumption via social media and politically motivated skepticism**
Subpopulation(s) most affected	▪ Hispanic and African American communities ▪ Elderly patients ▪ Immigrant/refugee communities ▪ People with low education ▪ Political conservatives ▪ Religious communities	▪ Anti-vaccination population ▪ Political conservatives ▪ Hispanic and African American communities ▪ Indigenous communities
Related or overlapping barriers	▪ Primary news consumption via social media and politically motivated skepticism ▪ Vaccine hesitancy ▪ Perceived underrepresentation of minorities in clinical trials	▪ Vaccine hesitancy ▪ COVID-19 vaccine safety concerns
Potential solutions	▪ Endorsement/promotion of COVID-19 vaccines by local healthcare professionals, including PCPs ▪ Partner with local leaders and community-based organizations	▪ Endorsement/promotion of COVID-19 vaccines by faith-based organizations ▪ Endorsement/promotion of COVID-19 vaccines by friends and family

While many respondents believed concerns specific to COVID-19 vaccine safety may resolve naturally with a successful vaccination rollout, medical mistrust and generalized vaccine hesitancy were expected to remain persistent over time, particularly for young adults, African American and Hispanic populations, and populations with low educational attainment. Respondents cited partnerships with community-based leaders and organizations that promoted messaging from locally trusted individuals as offering the most viable path to diminishing the impact of medical mistrust and general vaccine hesitancy among affected communities.

Politically motivated skepticism was considered the least likely barrier to resolve over time, affecting anti-vaccination, politically conservative populations, as well as Hispanic and African American and indigenous populations. This barrier also presents a significant ongoing challenge to reaching herd immunity. Though respondents were skeptical of efforts to mitigate this particular motivator of skepticism towards COVID-19 vaccination, they cited endorsement and promotion of COVID-19 vaccines by faith-based organizations and patients’ friends and family as potential solutions (see Table 2).

### Single-dose vaccination appears capable of mitigating certain key access and equity barriers

**Fig. 4:**
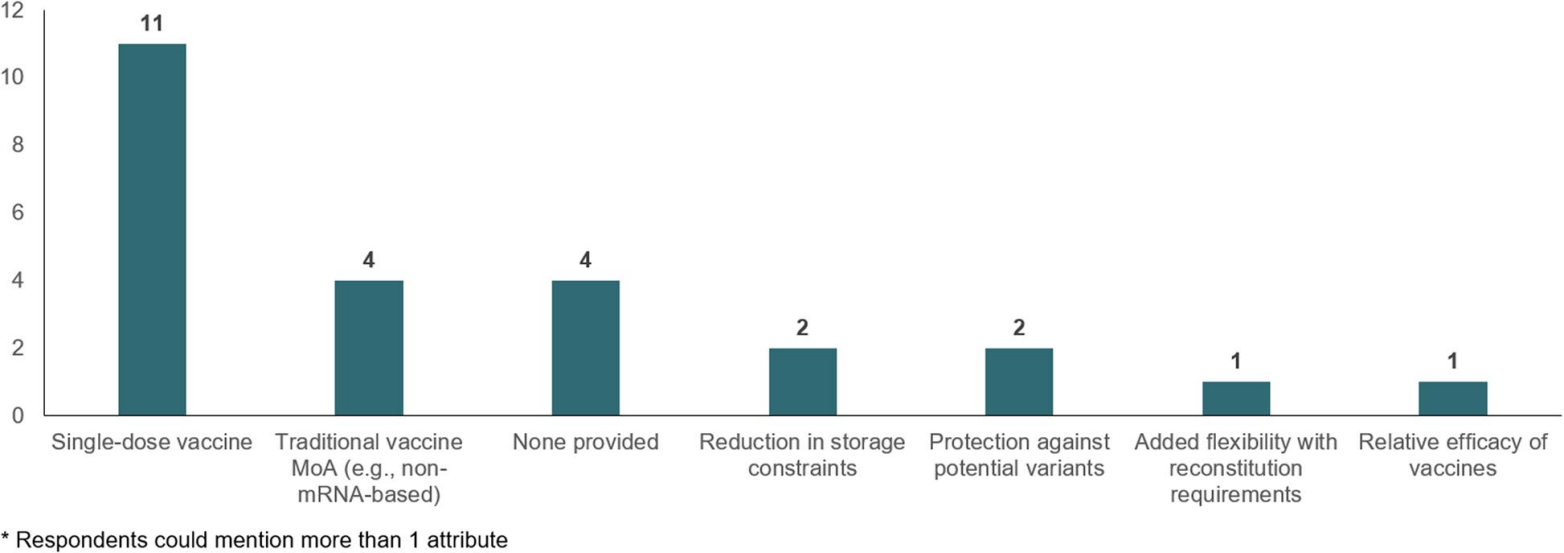
COVID-19 vaccine attributes addressing access and equity barriers (number of mentions*, *n* = 15 interviews)

When asked whether any COVID-19 vaccine attributes may mitigate barriers to access and equity, respondents most frequently mentioned a single-dose vaccine as having potential to counter disruption caused by certain structural barriers (see Fig. 4). Single-dose vaccines were thought to be particularly well-suited for vaccinations provided at patients’ homes, considered particularly important for harder-to-reach patients such as home-bound elderly, rural, and transient populations. Some respondents also noted that non-mRNA-based vaccines may reduce vaccine hesitancy caused by skepticism towards the novel mRNA platform used for some vaccines, which some patients viewed cautiously due to proliferation of misinformation on mRNA vaccine technology.

## Discussion

Stakeholders’ predictions that most of the structural and logistical barriers to access and equity in early stages of the COVID-19 vaccine rollout, such as inadequate staffing or complex eligibility tiering systems, would abate relatively quickly, have largely been proven accurate. While these barriers presented significant challenges to vaccination at the time stakeholders were interviewed in April and May of 2021, many key barriers have been resolved through proliferation of vaccination sites, provider and distributor experience, and vaccination mandates.

By contrast, attitudinal barriers to vaccine access and equity, such as generalized vaccine hesitancy, medical mistrust and politically motivated skepticism, were expected to be relatively persistent and indeed appear to present the most significant challenges to reaching herd immunity. If these barriers undermine access and equity for current and future booster shots they are likely to continue to present obstacles to ongoing COVID-19 prevention efforts. A subset of these barriers pertains to patient attitudes towards vaccines and medical care, which appear to present varying degrees of likelihood of resolution over time depending on their primary attitudinal motivation as specific, generalizable, or identity-linked in affected populations. Vaccine hesitancy that is solely driven by specific concerns about the COVID-19 vaccine development process or side effect profile, for example, may present a significant limitation to patient demand in the near-term. However, as stakeholders expected medical mistrust and politically motivated skepticism to remain relatively persistent over time, the degree to which these barriers overlap in patients with safety concerns may lead to continued depression of demand with few strategies beyond refinement of strategic messaging offering a path towards resolution.

Given the inherent challenges in resolving these attitudinal and demand-driven barriers, focusing on resolution of any lingering structural or logistical challenges may present a more effective strategy to target communities with lagging vaccination rates. For example, patients living in rural areas, particularly those who are not subject to employer or school mandates, may require additional efforts from providers to accommodate their willingness to receive a COVID-19 vaccine as they continue to lag the broader population in vaccinations. As these structural barriers are thought to affect primarily elderly, migrant, and lower-income populations, their persistence can be expected to perpetuate inequitable COVID-19 vaccine distribution if left unresolved. Mobilizing providers to travel to these patients’ homes or work sites may offer a viable path to closing the “last mile” gap and accommodate these patients’ willingness to receive a COVID-19 vaccine. Providers, community partners, and advocates suggested that vaccines most likely to enable this approach would be single-dose, avoiding logistical challenges expected in securing a follow-up appointment, and would carry minimal storage and portability restrictions to maximize mobility.

## Conclusion

Given universal availability of COVID-19 vaccines in the US, challenges to vaccinating a greater share of patients center on populations experiencing significant barriers to vaccine access and equity. Our study suggests that while these patients have faced an array of structural and attitudinal barriers, many structural barriers can be expected to resolve over time while attitudinal barriers such as politically motivated skepticism are expected to continue persisting into the future and present significant obstacles to reaching herd immunity. While these attitudinal barriers will likely need to be addressed through local communication campaigns, patients still affected by certain structural barriers may benefit greatly from vaccination programs that provide delivery and administration of COVID-19 vaccines at patient homes or places of work.

## Data Availability

The datasets generated and/or analyzed during the current study are not publicly available as we do not have consent from participants to share the transcripts of the blinded study interviews but are available from the corresponding author on reasonable request.

## Abbreviations

CDC: Centers for Disease Control and Prevention
KFF: Kaiser Family Foundation
TLR: Targeted literature review
